# Warfarin cessation is non-essential in patients undergoing total knee arthroplasty—a case-control study

**DOI:** 10.1186/s13018-015-0153-4

**Published:** 2015-01-28

**Authors:** Alfred Phillips, Michael Dan, Nathan Schaefer, Raymond Randle

**Affiliations:** Gold Coast University Hospital, Southport, QLD 4215 Australia; Maitland Hospital, Maitland, NSW 2320 Australia; John Flynn Private Hospital, Tugun, QLD 4224 Australia

**Keywords:** Total knee arthroplasty, Anticoagulation, Warfarin, Continuation, Cessation, Bridging, Complications

## Abstract

**Background:**

Warfarinised patients frequently present for total knee arthroplasty (TKA). Current practice of heparin ‘bridging’ is potentially cumbersome and hazardous. The research question is if cessation of warfarin is necessary for TKA.

**Methods:**

The study design was a retrospective case–control series of 61 warfarinised patients and 61 control patients undergoing TKA. TKA was performed by the senior author using a medial parapatellar approach without tourniquet. The target perioperative international normalised ratio (INR) for warfarinised patients was 2–2.2. Primary outcomes were changes in haemoglobin, transfusion requirements and complication rates.

**Results:**

There was no statistically significant difference between control and warfarin group in mean perioperative Hb (g/L) (pre-op 140 vs 141, day 0 115 vs 115, day 1 108 vs 111, *P* = 0.63), transfusion rates (14.75% vs 9.83%, *P* = 0.58), total complication rate (9.8% vs 9.8%, *P* = 0.75), demographics, range of motion or length of stay. There was a statistically significant higher use of the re-infusion drain in the warfarinised group (47.5% vs 24.6%, *P* = 0.014).

**Conclusion:**

This study supports the hypothesis that warfarin cessation is non-essential in patients undergoing TKA. This data is applicable to a patient group using re-infusion drains. Limitations of this study are typical of a small non-controlled observational study.

## Introduction

Total Knee arthroplasty (TKA) is a reliable treatment for end-stage arthritis. Osteoarthritis is the main aetiology of arthritis requiring TKA [1]. The incidence of osteoarthritis increases with age as do the relative comorbidities of the patient [2]. Common comorbidities include atrial fibrillation, venous thrombosis and valve replacement and these commonly require anticoagulation therapy, traditionally warfarin [3]. The Australian National Joint registry data demonstrates a 4.1% increase in TKA in 2012 from 2011 and a 92.4% increase since 2003 [1]. This is in keeping with a worldwide trend [4,5]. With an ageing population and a corresponding increase in the incidence of TKA, it is safe to assume an increasing proportion of patients who present for TKA will be on warfarin.

Warfarin exerts its anticoagulant affect as a vitamin K antagonist that inhibits clotting factors 2, 7, 9 and 10. This results in increased prothrombin time via impaired formation of fibrin. Conversely, proteins C and S are inhibited, which provides an initial pro-thrombotic effect. Warfarin is variably metabolised by the liver subject to patient factors such as genetics, diet and medications. The therapeutic window of warfarin is monitored via the international normalised ratio (INR). It has a half life of approximately 36 hours [6].

To manage the increased bleeding risk in patients undergoing TKA, warfarin is traditionally stopped 5 days pre-operatively. Patients are then assessed into low- and high-risk categories, with bridging anticoagulation in the form of low molecular weight heparin or unfractionated heparin recommended for higher risk patients [7].

The cessation of anticoagulation has been associated with a 1%–3% incidence of cerebrovascular events in specific populations of patients, resulting in significant morbidity and mortality [8,9]. There is also the concern that a rebound hypercoagulability on cessation can lead to increased risk of thrombotic events due to re-initiating warfarin [10,11]. Bridging therapy increases post-operative stay and healthcare costs as warfarin is restarted [12]. Recently, Simpson et al. found that bridging anticoagulation therapy for warfarinised patients undergoing TKA was associated with an increased risk of prolonged wound drainage, superficial infection, deep infection, washout and revision surgery [13].

In an effort to decrease risks and costs, studies have explored the safety of continuing warfarin perioperatively [9,14-17]. Rhodes et al. [18] and Chana et al. [19] suggest that cessation of warfarin perioperatively in patients undergoing TKA is unnecessary. These retrospective case–control studies including 38 and 24 patients in each group, respectively, are limited by low numbers. Our aim was to expand upon current literature and show that warfarin can be continued safely and effectively within its therapeutic range in patients undergoing TKA.

## Methods

The study design was a retrospective observational case–control series that required low-risk ethics approval and relevant data was obtained from the Health Information Services Unit. The senior author (RR) maintains lists of all his patients undergoing procedures while on any form of anticoagulation. The hospital reference numbers of the patients that underwent TKA while remaining on warfarin were used to locate charts and gather de-identified data. Data was extracted from inpatient notes, pathology, and operation and anaesthetic reports.

The inclusion criteria for the warfarin group of the study were long-term warfarinised patients (minimum of 6 months duration with stable INR) who underwent TKA by the senior author. No patients were excluded due to increased bleeding risk, high BMI, more complex TKA or at high risk of complications from general anaesthesia during the study period. Sixty one warfarinised patients were included in the study. The target INR range for the warfarinised group was 2–2.2 on the day of surgery. A total of 61 age- and gender-matched control subjects were chosen from the senior author’s booking diary during the same time period as the study group. The only information visible in the diary was the control patient’s name, date of birth and hospital reference number. The control subject age range was 6 months older or younger than the study subject. Patients were not matched for BMI but there was no statistically significant difference in BMI (29.7 kg/m^2^ for the control group vs 30.9 kg/m^2^ for the warfarin group (*P* = 0.23). Inclusion criteria for control subjects were age- and gender-matched patients undergoing TKA who were not on warfarin.

The study period included a consecutive cohort of warfarinised patients undergoing TKA from the time the senior author initially used this method of perioperative anticoagulation up the time of data collection. All warfarinised patients were on warfarin as lone anticoagulation. Six control patients were on aspirin for primary prevention of cardiovascular disease prior to surgery. Aspirin was ceased in all of these patients pre-operatively. All non-steroidal anti-inflammatory drugs were ceased 7 days pre-operatively in both study groups to optimise renal perfusion.

All warfarinised patients received a general anaesthetic. Control patients either received general or spinal anaesthetic informed by the anaesthetist and patient preference. All 122 patients were implanted with DePuy PFC implants between 2010 and 2013. With meticulous haemostasis, a medial parapatellar approach was used without tourniquet (not inflated at any stage during the procedure). All wounds were closed in ‘water-tight’ layers with the knee flexion with interrupted polydioxanone (PDS) and Monocryl to skin. There was no use of tranexamic acid (TXA), bipolar sealers or any other haemostatic techniques besides electrocautery during surgery. This cohort of patients was from a period before TXA was in mainstream use. All patients had re-infusion drains inserted intra-operatively and standardised pathology collected. All drains were removed day 1 post-operatively. All patients were able to full weight bear immediately post-operatively and underwent a standardised physiotherapy rehabilitation protocol commencing day 1 post-operatively. The senior author made all decisions regarding post-operative transfusion using the National Health and Medical Research Council (NHMRC) transfusion guidelines depending on the volume of the blood loss, fluid status, Hb concentration and the patient’s clinical condition. Patients who were symptomatically anaemic with Hb of <80 or <100 g/L in those with a documented history of end-organ atherosclerotic disease (i.e., ischaemic heart disease, chronic renal failure) were transfused red blood cells.

Warfarin dosing in the warfarin group was titrated using INR in an attempt to achieve a perioperative range of 2–2.2. The INR was checked within 48 h pre-operatively in all warfarinised patients. Patients were counselled pre-operatively about the effects of diet on INR and encouraged not to make any significant changes to their diet perioperatively. Patient diet was not formally modified or monitored at any stage of care. Control subjects were commenced on rivaroxaban (10 mg once daily) as a lone anticoagulation agent in the immediate post-operative period. All patients used the same mechanical venous thromboembolism (VTE) prophylaxis while inpatients.

All patients received a standardised pre-operative assessment including basic blood tests and coagulation profiles by the senior author. Standardised post-operative outcome data from clinical reviews at 2 weeks, 6 weeks and 6 months were used to assess medium-term outcomes for all patients. The minimum length of follow-up for the study was 6 months. This timing corresponded to when patients were first discharged back into the care of their local doctor. The average follow-up was 8.5 months for the study group (range 6 to 15 months) and 9 months for the control group (range 6 to 13 months). All outcomes and adverse events were included up until the time of discharge or if the patient was seen again for a related matter on an ‘as needed’ basis.

Statistical analysis was performed using SPSS version 21 for Windows (SPSS Inc., Chicago, IL). Variables were summarised by the mean, standard deviation (SD), 95% confidence intervals or frequency. Chi-square tests were used to compare categorical variables. Independent sample *T* tests were used to compare quantitative variables for two groups. A repeated measures analysis of variance (ANOVA) was performed to test the hypothesis that warfarin is associated with different Hb levels across time compared to controls. Statistical significance was set at *P* < 0.05 for all tests. A post hoc power analysis was performed with observed values from the data collected. The sample size used in this study (*n* = 61 in each group) yielded approximately 8% power to detect the effect size 0.2% as being statistically significant (*P* < 0.05). This was based on a mean of 108 g/L (SE = 1.7) in controls and a mean difference of ~2.6 g/L between control and study group at day 1.

## Results

There was no statistically significant difference in patient age, gender or pre- and post-operative range of motion between the warfarin and control groups. There was no statistically significant difference in BMI between the two groups (29.7 kg/m^2^ for control group vs 30.9 kg/m^2^ for the warfarin group, *P* = 0.23). There was no statistically significant difference in surgical time between the two groups. The mean operative time for the control group was 56 min vs 54 min for the warfarin group (*P* = 0.31). The mean pre-operative INR in the warfarinised group was 2.0 (range 1.9 to 2.3). The most common indication for warfarinisation of patients in this study was a history of atrial fibrillation and venous thromboembolism shown in Table 1.

**Table 1:** **Frequency distribution of indications for warfarinisation**

**Indication for warfarin in the study group**	**Number (%) of the study group**
History of atrial fibrillation	33 (54.1)
Previous venous thromboembolism	17 (27.9)
Cerebrovascular accident	3 (4.9)
Myocardial infarction	2 (3.3)
Valvular heart disease	2 (3.3)
Severe peripheral vascular disease	1 (1.6)
Paroxysmal atrial arrhythmia and pacemaker	1 (1.6)
Mechanical heart valve	1 (1.6)
Ischaemic heart disease	1 (1.6)

**Figure 1:**
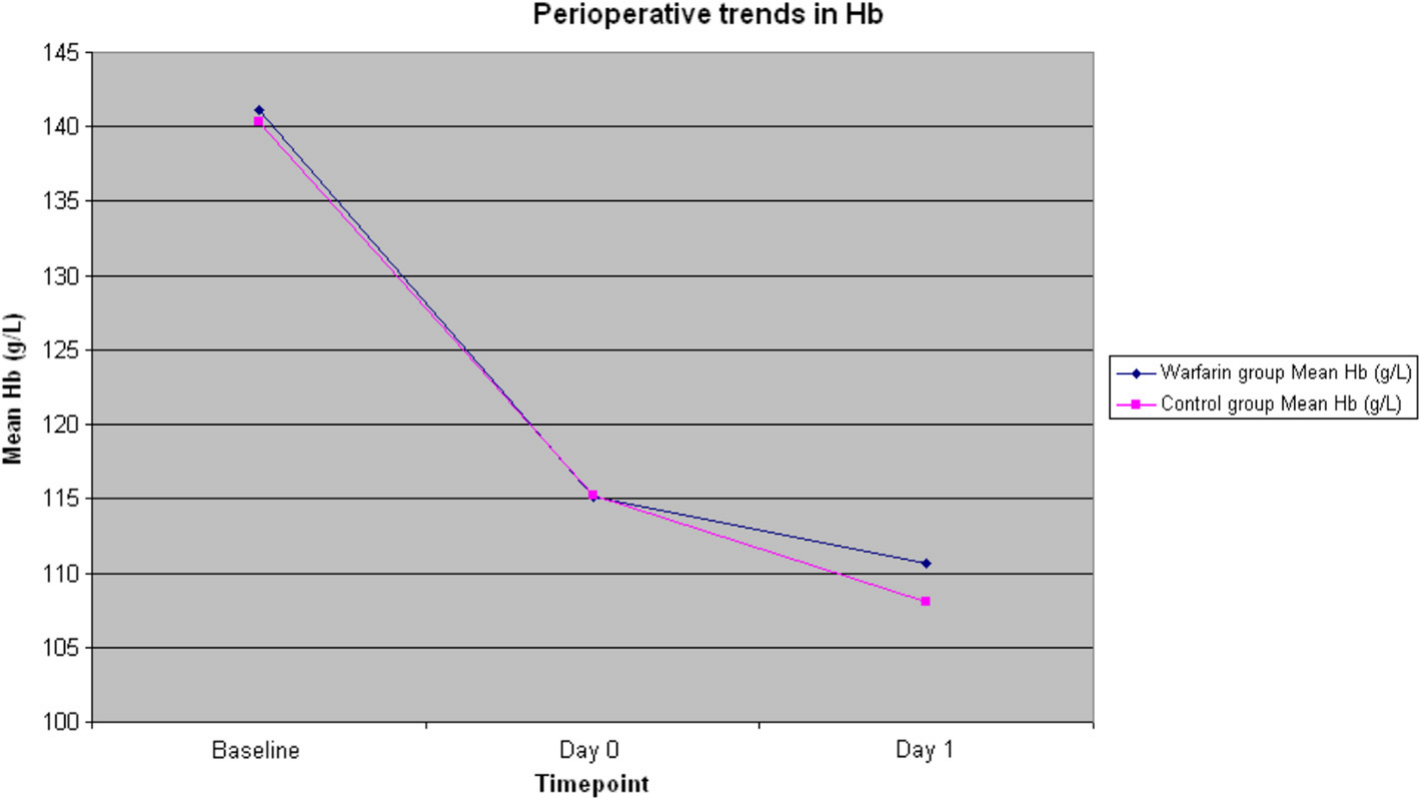
**Perioperative trends in haemoglobin.**

There was no statistically significant difference between the control and warfarin group in the mean perioperative haemoglobin (pre-op 140 vs 141 g/L, day 0 115 vs 115 g/L, day 1 108 vs 111 g/L *P* = 0.63) as shown in Figure 1.

There was no statistically significant difference in total transfusion rates (14.75% vs 9.83%, *P* = 0.58) as shown in Table 2.

**Table 2:** **Post-operative transfusion requirements**

	**Control group (** ***n*** **= 61)**	**Warfarin group (** ***n*** **= 61)**	***P*** **value**
Number of PRBCs transfused during admission			
0	53	55	
1	1	0	
2	6	3	
3	1	1	
4	0	1	
5	0	1	
6	1	0	
Total % of patients requiring transfusion of PRBCs	14.75%	9.83%	*P* = 0.58
Total number of units PRBCs transfused	21	14	

There was a statistically significant higher use of the re-infusion drain in the warfarinised group (47.5% vs 24.6 %, *P* = 0.014) as shown in Table 3. The mean INR for the warfarin group was 2.0 pre-operatively, 2.5 on days 1 and 2 and 2.3 on day 3 post-operatively.

**Table 3:** **Post-operative use of re-infusion drains**

	**Control group (** ***n*** **= 61)**	**Study group (** ***n*** **= 61)**	***P*** **value**
Volume re-infused via Stryker re-infusion drain (mL)			
0	46	32	
100–200	1	6	
200–300	8	9	
300–400	4	7	
400–500	1	3	
500–600	0	2	
600–700	0	2	
700–800	0	0	
800–900	1	0	
Use of re-infusion drain (%)	24.6	47.5	*P* = 0.014

There was no statistically significant difference in the total complication rate (9.8% vs 9.8%, *P* = 0.75). A warfarinised, immunosuppressed, morbidly obese patient with rheumatoid arthritis developed a septic TKA. Pre-operative counselling addressed her significantly increased risk of infection. She underwent two-stage revision and made a full recovery with a range of motion of 0°–105°.

A warfarinised patient was re-admitted day 14 post-operatively with a haemarthrosis secondary to a supratherapeutic INR of 8. The haemarthrosis was treated conservatively with ice and elevation while his INR was corrected. He was discharged day 3 post re-admission and made a good recovery with a range of motion of 10°–115°.

A warfarinised patient with a history of lower gastrointestinal tract bleeds was re-admitted day 8 post-operatively with malaena and Hb of 78 g/L. The patient was transfused two units of packed red bloods cells and four units of fresh frozen plasma. An upper endoscopy and colonoscopy did not identify the source of bleeding. The malaena resolved and the patient made a good functional recovery with a range of motion of 3°–110°.

A warfarinised patient with long-standing mild cognitive impairment fell out of bed during an episode of acute confusion post-operatively. They sustained a superficial wound dehiscence that required closure under general anaesthetic. The wound healed well; however, poor compliance post-operatively limited recovery, resulting in a range of motion of 45° to 105°.

A warfarinised patient with a history of ischaemic heart disease suffered a non-ST elevated myocardial infarction (NSTEMI) day 3 post-operatively in the setting of analgesic nephropathy. The patient was treated with a GTN infusion and PCI where they were found to have 50% stenosis of their left anterior descending (LAD) coronary artery. A good functional recovery achieved a range of motion 0°–115°.

A warfarinised patient with an extensive past medical history of cardiovascular events suffered a TIA post-operatively. A carotid Doppler ultrasound showed a 50%–69% carotid stenosis. The patient was treated conservatively and made a good recovery with a post-operative range of motion of 20°–105° (pre-operative ROM 20°–95°).

A control patient was readmitted 3 weeks post-operatively with a 3-day history of malaena and haemoglobin of 77 g/L. The patient underwent endoscopy and was found to have duodenal erosions. The patient was transfused three units of packed red blood cells. The patient recovered well and achieved a range of motion of 5°–90°.

A control patient developed anaemia unresponsive to a total of six units of packed red bloods cells. The patient had a history of severe peripheral vascular disease, left below-knee amputation (BKA), polymyalgia rheumatica on oral steroids and chronic obstructive pulmonary disease (COPD) on home oxygen. Day 7 post-operatively, the patient underwent gastroscopy that identified distal gastritis that was treated with a proton pump inhibitor. The patient also developed bilateral pulmonary emboli on CT pulmonary angiogram at 8 weeks post-operatively. The patient was treated with aspirin and warfarin (with LMWH bridging). The patient had significant hamstring spasm and compliance issues post-operatively with a range of motion of 45°–100°. At last review, the patient declined to undergo manipulation under anaesthesia.

A control patient suffered a superficial wound dehiscence while flexing their knee day 3 post-operatively. The wound was closed under local anaesthetic and healed well. The patient made a good functional recovery with a range of motion at 0°–110°.

A control patient suffered a seizure in the immediate post-operative period in the recovery suite. The patient likely received an inadvertent intravenous dose of local anaesthetic for a regional block by the anaesthetic team. The patient had no hypotension or signs of decreased cardiac output. They received 5 mg of intravenous midazolam, which resulted in immediate cessation of seizure activity. The patient was transferred to the intensive care unit for observation and discharged to the general orthopaedic ward day 2 post-operatively with no long-term sequelae. The patient made a good post-operative recovery with a range of motion of 5°–110°.

A control patient developed post-operative confusion secondary to anaemia day 3 post-operatively. The patient had a normal CT head and a negative septic screen. They received three units of PRBCs and had a good long-term outcome with a range of motion of 5°–100°.

## Discussion

The results of this study support the hypothesis that cessation of warfarin in patients undergoing total knee arthroplasty is not necessary. Warfarin continuation was shown to be a safe and effective way of anticoagulating patients perioperatively. Two other studies (Rhodes et al. and Chana et al.) have investigated the continuation of warfarin in patients undergoing TKA [18,19].

Rhodes et al. and Chana et al. found no increase in the rate of haemorrhage for patients on continuous warfarin. In our study, of 61 warfarinised patients undergoing TKA, the mean pre-operative INR in the warfarinised group was 2.0 with a mean increase of 0.3 by day 3 post-operatively. The senior author designated the optimal perioperative INR range of 2–2.2. Chana et al. reported similar results with a mean pre-operative INR of 2.2 and a mean change in INR of 0.4 [19]. Rhodes et al. also reported a mean pre-operative INR of 2.1 and a mean change in INR of 1.2 [18].

There was no statistically significant difference in perioperative haemoglobin and total complication rates between warfarinised and control patients in our study. Both Chana et al. and Rhodes et al. also found that there was no statistically significant difference in complication rates in warfarinised patients undergoing TKA [18,19]. None of the warfarinised patients in our study suffered any embolic events such as VTE or CVA.

No statistically significant difference in the transfusion requirements of the two groups was demonstrated with 9.8% of warfarinised patients and 14.8% of control patient requiring blood transfusions. Both Chana et al. and Rhodes et al. also found that there was no statistically significant difference in transfusion requirements in warfarinised patients undergoing TKA [18,19].

All patients in our study had a closed suction drain that was combined with a collection-re-infusion system for post-operative blood recovery (Stryker CBCII). We chose to include this data due to the oxygen carrying capacity of the drain contents. Several studies have highlighted the merit of these systems in successfully decreasing allogenic transfusion requirements in total joint arthroplasty [20-24]. There was a statistically significant increase in autotransfusion requirements of warfarinised patients in our study. This was a significant finding given that there was no difference in perioperative Hb between the two groups. We recommend that our data is applicable to warfarinised patients undergoing TKA using a similar drain.

In our study, warfarinised and control patients were age- and gender-matched. Patient BMI and pre- and post-operative range of motion was recorded. There was good homogeneity of patient demographics between the two groups. There was no statistically significant difference in pre- and post-operative range of motion. The majority of patients in our study were warfarinised for either atrial fibrillation (AF) or previous VTE. In the setting of untreated AF, data from the Framingham studies has shown a 28.2% risk of CVA over an 11-year period [25]. Warfarinised patients with a history of transient ischaemic attack, diabetes or ischaemic heart disease have even greater risk of stroke when not anticoagulated [26]. Despite these risks, the American College of Cardiology maintain that warfarin can be ceased for up to a week without bridging for procedures that carry a risk of haemorrhage [27]. The group, along with most surgeons, advocate for individualised risk stratification when determining the most effective form of perioperative anticoagulation in this patient population [28].

A recent study by Simpson et al. has highlighted the increased incidence of post-operative complications when warfarinised patients are bridged with another agent for TKA [13]. In this study, 149 patients on warfarin pre-operatively were bridged with either low- or high-dose unfractionated heparin, low- or high-dose low molecular weight heparin, IV heparin or aspirin. There were significantly higher complication rates in this patient group compared to the control group with the bridged group at particularly high all-cause risk [1.8, (95% CI 1.15 to 2.35), *P* = 0.001]. There was also a significant higher incidence in prolonged wound drainage (26.8% of cases vs 7.3% of controls, *P* = 0.001); superficial infection (16.8% vs 3.3%, *P* = 0.001); deep infection (6.0% vs 0% *P* = 0.001); return-to-theatre for washout (4.7% vs 0.7%, *P* = 0.004) and eventual revision (4.7% vs 0.3%, *P* = 0.001). The limitations of the study by Simpson et al. were multiple contributing surgeons, the type of thromboprophylaxis in the control group was not standardised, the use of tourniquets and drains was not standardised and there was no uniform transfusion protocol. Despite these limitations, the findings by Simpson et al. are in keeping with our anecdotal experience and difficulty with bridging warfarinised patients. This prompted the senior author to first consider continuing his patients on warfarin perioperatively. There are currently two large multi-centre randomised control trials PERIOP 2 and BRIDGE assessing whether post-operative bridging reduces risk of VTE or increases morbidity.

The limitations of this study are typical of small, non-controlled, retrospective, observational studies. The number of warfarinised patients was low, which resulted in the study being relatively underpowered and the statistics described are susceptible to type II error. The recruitment of these patients was passive and therefore difficult to increase. The authors acknowledge that, despite being age- and gender-matched, the control subjects were manually selected from an operative diary. The senior author’s practice does not use electronic records, which made it difficult to overcome this issue. We also acknowledge that complications were compared as a total complication rate and we did not account for the severity of individual complications. The decision to continue warfarin perioperatively precludes patients from undergoing spinal anaesthesia due to the risk of epidural haematoma. The senior author has not had to cease warfarin for any patients undergoing TKA. Despite being the largest series of warfarinised patients undergoing TKA, our data does not provide sufficient evidence to recommend cessation of warfarin pre-operatively in more complex cases such as revision surgery. We recommend that all patients with increased but reducible perioperative risk should be medically optimised. While there are no absolute contraindications to general anaesthesia, valvular heart disease is a common contraindication to regional anaesthesia for which many patients are warfarinised. Current literature suggests that there is no increased risk of mortality, cardiac complications or VTE between spinal and general anaesthesia [29-31].

The strengths of this study are that it has a simple design and it is the largest published cohort of warfarinised patients undergoing TKA. It was a single-centre, single-surgeon study. All patients received the same implants and the senior author made all clinical decisions.

## Conclusion

The primary consideration for surgeons planning to perform TKA on chronically warfarinised patients is balancing the risks of thromboembolism against post-operative complications. This is challenging. In an attempt to mitigate risk of complications, patients have traditionally had warfarin withheld pre-operatively and bridged with a shorter acting form of anticoagulation. This strategy is potentially perilous for the patient and cumbersome for the clinician. In conclusion, the authors feel that continuation of warfarin is a safe and effective form of perioperative anticoagulation in patients undergoing TKA. Re-infusion drains should be used on warfarinised patients undergoing TKA. While acknowledging the limitations of this study, the findings contribute to the discrete body of literature that supports the notion that warfarin cessation is non-essential in patients undergoing total knee arthroplasty with concomitant use of a re-infusion drain.
